# Epidemiological and Clinical Characterization of Atopic Dermatitis in Dogs from Quito, Ecuador: Retrospective Analysis of Cases (2018–2025)

**DOI:** 10.3390/vetsci13040351

**Published:** 2026-04-03

**Authors:** Verónica Pareja-Mena, Daniela Flor-Dillon, Byron Puga-Torres, Anthony Loor-Giler, Luis Núñez

**Affiliations:** 1 CEVET, Centro de Especialidades Veterinarias, Alemania y Vancouver, Quito EC 170515, Ecuador; veronica_pareja@hotmail.com; 2 Facultad de Medicina Veterinaria y Zootecnia, Universidad Central del Ecuador, Jerónimo Leyton S/N y Gatto Sobral, Quito EC 170515, Ecuador; danielaflor95@hotmail.com (D.F.-D.); bpuga@uce.edu.ec (B.P.-T.); 3 Laboratorios de Investigación, Dirección General de Investigación, Universidad de las Américas (UDLA), Antigua Vía a Nayón S/N, Quito EC 170124, Ecuador; a.abel.loor.giler@gmail.com; 4 Facultad de Ciencias Veterinarias, Universidad de Buenos Aires, Av. Chorroarín 280, Buenos Aires 1427, Argentina; 5 Facultad de Ciencias de la Salud, Carrera de Medicina Veterinaria, Universidad de Las Américas, Antigua Vía a Nayón S/N, Quito EC 170124, Ecuador; 6 One Health Research Group, Facultad de Ciencias de la Salud, Universidad de Las Américas, Quito EC 170124, Ecuador

**Keywords:** canine atopic dermatitis, Ecuador, epidemiology, clinical characteristics

## Abstract

Canine atopic dermatitis (CAD) is a chronic, pruritic inflammatory disease that affects up to 15% of the global canine population. Its etiopathogenesis is multifactorial, involving genetic, immunological, environmental, and dietary factors. It is characterized by pruritus, erythema, alopecia, and secondary lesions, predominantly affecting the abdomen, extremities, and ears. This retrospective cross-sectional descriptive study analyzed 735 medical records of dogs diagnosed with CAD treated at the Veterinary Specialty Center (CEVET) in Quito, Ecuador, between January 2018 and July 2025. Demographic, clinical, housing, diet, and cohabitation data were collected and statistically analyzed using χ^2^ for qualitative variables and the Kruskal–Wallis test for quantitative variables, with post hoc analysis as appropriate. Additionally, pruritus severity was assessed using the Pruritus Visual Analog Scale (pVAS). A composite Clinical Severity and Distribution Score (CSDS) was also developed to classify disease severity. A multivariate logistic regression model was performed to identify factors associated with severe CAD. The results showed a predominance of CAD in adult dogs (84.2%) and purebred dogs (74.97%), with a slight majority being males (52.38%). Pruritus was the most frequent initial symptom (80.27%), with most cases presenting moderate-to-severe pruritus (pVAS 7–10). The most affected areas were the abdomen (24.49%) and forelimbs (17.68%). The geographical distribution showed a predominance of urban areas (88.84%) and cold climates (86.39%). Based on the CSDS, 53.2% of cases were classified as severe, 44.4% as moderate, and 2.4% as mild. Multivariate analysis revealed that grass exposure was significantly associated with severe CAD (OR = 1.78; 95% CI: 1.22–2.60; *p* = 0.003), while urban environment showed a non-significant trend toward increased severity (OR = 1.41; *p* = 0.071). Significant associations were identified involving sex and body weight, age and affected area, and temporal variations in the severity of pruritus, age group, and distribution of lesions. Among breeds, French Bulldogs, Standard Schnauzers, and Shih Tzus had the highest prevalence of CAD. These findings provide the first systematic epidemiological and clinical characterization of CAD in Ecuador, highlighting the role of environmental factors in disease severity and supporting the use of composite clinical scoring approaches in retrospective studies, thereby contributing to understanding of the disease and serving as a reference for early diagnosis, clinical management, and the development of preventive strategies.

## Simple Summary

Canine atopic dermatitis (CAD) is a chronic, pruritic, and inflammatory skin disease influenced by genetic, immunological, environmental, and dietary factors. This retrospective study analyzed 735 CAD cases diagnosed in Quito, Ecuador, between 2018 and 2025 and showed a higher prevalence in adult purebred male dogs compared with mixed-breed animals. The most frequently reported initial symptom was pruritus, with greater involvement of the abdomen and extremities. Urban dogs in cold climates were diagnosed more frequently. Significant correlations were identified between CAD and age, sex, breed, weight, and lesion location, laying the foundation for clinical management and future research in Ecuador.

## 1. Introduction

Canine atopic dermatitis (CAD) is a multifactorial inflammatory skin disease [1], characterized by chronic pruritus, affecting approximately 10–15% of the global canine population (*Canis lupus familiaris*). The disease is associated with a genetic and hereditary predisposition, leading to the production of immunoglobulin E (IgE) against environmental or food allergens [2,3]. CAD is considered one of the three most frequently diagnosed allergic diseases in veterinary practice and is characterized by frequent relapses, which contribute to its chronic course [4]. Diagnosis is based on the exclusion of other dermatological conditions, patient clinical history, and specific intradermal allergy testing, including IgE-based assays [5]. In addition to intradermal testing, serological assays for allergen-specific immunoglobulin E (IgE) are commonly used as complementary diagnostic tools to identify environmental allergens associated with canine atopic dermatitis [5].

The etiopathogenesis of CAD is multifactorial, involving genetic, environmental, dietary, and immunological factors, with various forms of diagnosis [6]. Persistent pruritus is the most relevant clinical sign of CAD, significantly compromising the quality of life of both the animal and its owner. Since there are no specific diagnostic tests, diagnosis is based on the exclusion of other causes of pruritus and the application of clinical criteria, which consider the age of onset, breed, and lesion distribution [7].

Clinical signs include moderate-to-severe pruritus, erythema, papules, macules, alopecia, and secondary lesions, which are mainly distributed on the ears, axillae, abdomen, inguinal region, and perianal area [8]. Alterations in skin pH and barrier function promote the proliferation of opportunistic microorganisms such as *Staphylococcus pseudintermedius* and *Malassezia pachydermatis*. These secondary infections aggravate lesions and are associated with clinical manifestations such as external otitis, hyperpigmentation, lichenification, and excoriations [5,8,9]. However, significant clarification is still required regarding host-microbiome interactions and the impact of cutaneous dysbiosis on the initiation or aggravation of CAD [3,10].

The treatment of CAD is aimed at controlling clinical signs and preventing relapses. Therapy includes the use of systemic or topical glucocorticoids, cyclosporine, oclacitinib, and new Janus kinase (JAK) inhibitors, such as ilunociitinib, which acts by regulating the inflammatory response and pruritus, as the same with luteolin [11,12,13,14]. However, prolonged use of these drugs may lead to adverse effects, requiring careful clinical monitoring. In this context, topical therapy plays an essential role in restoring skin barrier function. The application of essential fatty acids, unsaturated fatty acids (especially omega 3 and 6), short-chain fatty acids (acetic, propionic, and butyric), ceramides, phytosphingosine, and cholesterol helps restore the lipid balance of the stratum corneum and reduce the need for systemic treatments [15,16]. At the same time, hypoallergenic diets and nutritional supplements complement the comprehensive management of the patient [17,18]. Pain relief treatments could also be used including cannabinoids [19], as well as the clay mineral bentonite as a preventive measure for cases of *Staphylococcus pyoderma* [20,21]. In recent years, several novel therapeutic approaches have been explored for allergic skin diseases in companion animals including targeted immunomodulatory therapies, biological agents, and new Janus kinase inhibitors that modulate cytokine signaling pathways involved in pruritus and inflammation [11,22,23].

Among the most promising strategies is allergen immunotherapy (AIT), considered the only etiological treatment capable of modifying the course of the disease. This involves the controlled administration of increasing doses of the allergen to induce immune tolerance and reduce hypersensitivity [24,25,26]. In addition, regenerative and immunomodulatory approaches using mesenchymal stem cells derived from the canine amniotic membrane [27,28]. Advances in comparative immunology have revealed significant similarities between CAD and human atopic dermatitis, encompassing common features such as genetic predisposition and spontaneous disease development [29,30]. Likewise, mutations have been identified in genes that encode structural proteins such as filaggrins, which are essential for skin barrier [5,25,31]. Based on this background, and since there are no studies in Ecuador that systematically document the epidemiological and clinical characteristics of CAD, this research allows us to establish the frequency of the disease in different breeds, age, sex, and regions of the country, contributing to the global epidemiology of CAM. In addition, it makes it possible to identify common clinical patterns, which is particularly important because it will provide an unprecedented database that will contribute to the recognition of CAD as a dermatological health problem in the Ecuadorian canine population and in other countries that have similar characteristics to those presented here. The results will serve as a reference for clinical practice, early diagnostic guidance, rational selection of treatments, and the design of future research aimed at the prevention and control of this disease.

## 2. Materials and Methods

This study was conducted using an observational, descriptive, and retrospective design based on dogs diagnosed with canine atopic dermatitis (CAD). Diagnosis was established after excluding other dermatological conditions, reviewing the patients’ medical history, and performing specific intradermal tests. The dogs were observed between January 2018 and July 2025 at the dermatology department of the Centro de Especialidades Veterinarias (CEVET), a leading veterinary center in the city of Quito, Pichincha province, Ecuador. CAD was diagnosed based on the previously described criteria [5] relying on clinical history, exclusion of other pruritic dermatological diseases (including ectoparasitic infestations, dermatophytosis, and secondary infections), and the application of established clinical criteria such as those proposed by Favrot et al. [32].

Inclusion criteria comprised dogs diagnosed with CAD whose medical records contained information based on origin, geographical distribution, climate, sex, purebred or mixed breed status, age in months, age group, internal and external deworming, first sign, degree of pruritus, most affected body area, diet, cohabitation with other pets, exposure to grass, and type of housing (Supplementary Materials, Table S1).

**Figure 1:**
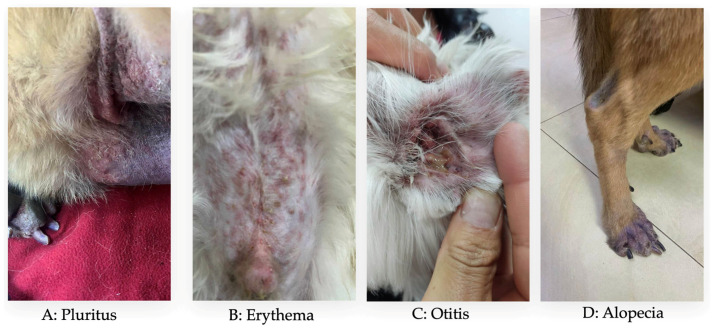
Predominant clinical sign in several dogs with CAD in Ecuador.

**Figure 2:**
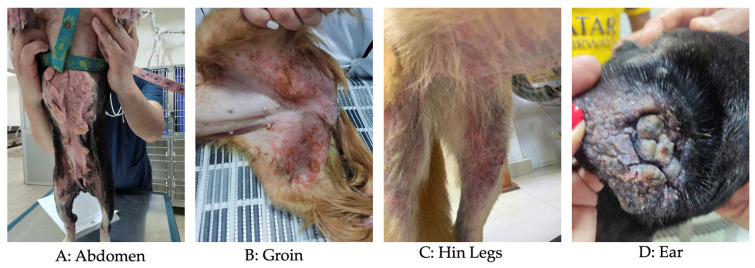
Anatomical regions affected in several dogs with CAD in Ecuador.

The intensity of pruritus was evaluated using the Pruritus Visual Analog Scale (pVAS), a validated tool widely used in veterinary dermatology to assess the severity of pruritus reported by dog owners [33,34]. This scale is continuous, ranging from 0 to 10, where 0 indicates no pruritus and 10 represents the most severe possible pruritus. For analytical purposes, pVAS scores were developed and categorized as an ordinal variable defined on a scale of 1 to 3 where 1 = mild (0–3 pVAS), 2 = moderate (4–6 pVAS) and 3 = severe (7–10 pVAS). This transformation reduced inter-rater reliability and facilitated statistical analysis. Lesion severity in CAD is commonly assessed using standardized scoring systems such as the Canine Atopic Dermatitis Extent and Severity Index (CADESI) or the Canine Atopic Dermatitis Lesion Index (CADLI) [35,36]. However, due to the retrospective nature of the dataset and the absence of detailed lesion scoring across multiple anatomical regions, these indexes could not be calculated. Therefore, a composite clinical score, named the Clinical Severity and Distribution Score (CSDS), was developed to characterize disease severity based on available variables. The CSDS integrates pruritus severity (based on categorized pVAS), predominant clinical sign and the extent of skin lesions. The predominant clinical sign reported by the owner at the time of the consultation (e.g., pruritus, erythema, otitis, alopecia or odor) were recorded and categorized into an independent ordinal variable called symptom score (Figure 1). This score was based on a scale of 1 to 3 where 1 indicated a single symptom, 2 indicated three symptoms, and 3 indicated more than three symptoms. This variable was constructed by assigning values according to the clinical relevance of the initial sign in the context of CAD, allowing the incorporation of early clinical presentation as an additional dimension of severity. The extent of skin lesions was assessed by the number of anatomical regions affected (e.g., Abdomen, Groin, Hin legs, Ear, etc.) in each animal (Figure 2). From this information, an ordinal variable called area score was generated, defined a scale of 1 to 3 where 1 indicated involvement of a single region, 2 indicated involvement three regions, and 3 indicated involvement more than three affected regions. This approach allowed for the standardized quantification of the body distribution of lesions. Based on these three clinical dimensions, namely pruritus intensity (pvas score), predominant clinical sign (symptom score), and extent of skin lesions (area score), a composite index CSDS was constructed. This score was calculated as the sum of the three variables (CSDS = pVAS score + symptom score + area score), with a theoretical range of 3 to 9 points, where 3–4 represents mild, 5–7 moderate, and 8–9 severe (Supplementary Materials, Table S1). This index allowed for the integration of the owner’s perception, the initial clinical manifestation, and the anatomical extent of the lesions into a single measure of clinical severity. The CSDS was used as the dependent variable in statistical analyses, including multivariate regression models, to identify factors associated with the clinical severity of the disease in the studied population.

No personal or confidential information from caretakers was used. Medical records that did not contain this information were excluded. In total, 735 patients diagnosed with CAD were included, from Pichincha (717), Chimborazo (1), Carchi (1), Imbabura (2), Manabí (4), Orellana (1), Santo Domingo de los Tsáchilas (4), and Tungurahua (5). In terms of breed, the animals were classified as mixed breed (184 animals) or purebred (551 dogs), including French Bulldogs, Standard Schnauzers, Shih Tzus, Yorkshire Terriers, German Shepherd Dogs, West Highland White Terriers, Golden Retrievers, Labrador Retrievers, English Bulldogs, Poodles, Pitbulls, Pugs, Pekingese, Old English Sheepdogs, among others. Sample size was calculated assuming an expected CAD prevalence of 50%, with a desired absolute precision of 5% and a confidence level of 95%, using the formula for estimating sample size in observational studies, following the principles described for sample size estimation in observational studies [37]:

$$n=\frac{Z^{2}\cdot p\cdot (1-p)}{d^{2}}$$

*where the following definitions are used:*

- *n corresponds to the required sample size;*
- *Z^2^ is the value of the standard normal distribution for a 95% confidence level (Z = 1.96);*
- *p is the expected prevalence of the disease (0.50);*
- *d is the desired absolute precision (0.05).*

### Statistical Analysis

The database was constructed from all the result reports issued by CEVET, which were consolidated in a Microsoft Excel spreadsheet. Using disease frequency tables, prevalence was determined using Prevalence = (No. of positive cases)/(Total size of the assessed population). Prevalence was estimated for the following variables: ambient temperature category (cold or less than 15 °C, semi-temperate between 16 and 19 °C, temperate 20–25 °C, hot greater than 25 °C), based on the IPCC [38]; sex (male or female), body weight (kg), breed status (purebred or mixed breed), age group [puppy (1–12 months); adult (13–132 months); geriatric (133 months or older)] [39]; internal and external deworming (yes/no).

The analysis also included the first clinical sign reported by the guardian (alopecia, bad odor, erythema, otitis, pruritus); the degree of pruritus according to the Pruritus Visual Analog Scale [34]; the most affected visible lesion area (abdomen, armpits, back, croup, ears, eyelids, front legs, hind legs, mouth, neck, tail, thorax); diet type [Barf, Homemade Food, Mixed (Homemade food plus dry dog food), Premium, Standard, Super Premium], cohabitation with other pets (cats, dogs, dogs and cats, unique pet): exposure to grass (yes or no); and type of housing (apartment with lawn, apartment without lawn, house with lawn, house without lawn).

Statistical comparisons between qualitative variables (internal and external deworming, sex, breed, year of study, age, area affected by CAD, pruritus, geographic distribution) were performed using the chi-square test (χ^2^). When significant differences were determined, post hoc pairwise comparisons of proportions with Bonferroni correction were applied. In the case of quantitative variables (age in months and body weight), the Kolmogorov–Smirnov test was applied to determine the normality of the data; as there was no normal distribution (*p* value: <0.05), the non-parametric Kruskal–Wallis test was used for comparison in relation to qualitative variables. When there were significant differences, the Dunn test with Bonferroni correction was used as a post hoc analysis.

Additionally, a multivariate logistic regression analysis was performed to identify factors associated with severe disease presentation. The dependent variable was defined as severe CAD (CSDS ≥ 8), while independent variables included age, sex, environmental exposure (urban vs. rural), and contact with grass. Odds ratios (OR) and 95% confidence intervals (CI) were calculated. Model selection was based on clinical relevance and data distribution, ensuring parsimony and minimizing overfitting.

In all cases, RCran software version 1.2.5019 (RStudio Inc. Boston, MA, USA) and its RStudio platform version 2024.04.2+764 were used with a significance level of 0.05.

## 3. Results

Between January 2018 and July 2025, a total of 735 patients who met the established inclusion criteria were enrolled, and the results are based on this number of cases.

### 3.1. Demographic Distribution of CAD

Table 1 summarizes the demographic distribution of CAD patients treated at CEVET, where patients were from eight provinces of Ecuador (Carchi, Chimborazo, Imbabura, Manabí, Orellana, Pichincha, Santo Domingo de los Tsáchilas, and Tungurahua). Regarding annual distribution, between January 2018 and July 2025. Looking at the number of patients in each year, between 2018 and 2020, 3.4% (25/735), 3.54% (26/735), and 3.81% (28/735) of patients were treated, respectively. After the pandemic, in 2021, 20.68% (152/735) of cases were treated, in 2022 18.91% (139/735), in 2023 17.14% (126/735), in 2024 24.22% (178/735), and in the first half of 2025 8.3% (61/735).

As the Veterinary Center is located in Quito, Pichincha, 97.55% (717/735) came from this province; likewise, 86.39% (652/735) live in a cold climate, while 10.88% (82/735) live in a temperate climate, 2.05% (15/735) in a semi-temperate climate, and 0.68% (5/735) in a warm climate. In terms of geographical distribution, 88.84% (671/735) of the animals diagnosed with CAD come from urban areas, while 11.16% (84/735) come from rural areas.

Regarding environmental exposure, 64.35% (473/735) of the dogs have contact with grass, while 35.65% (262/735) do not. Regarding housing, 58.78% (432/735) of the animals live in houses with access to grass, 19.18% (141/735) in apartments without access to grass, 16.46% (121/735) in houses without access to grass, and 5.58% (41/735) in apartments with access to grass. Finally, 48.16% (354/735) of the animals live with other dogs, 46.94% (345/735) are the only pet, 4.49% (33/735) live with cats, and 0.41% (3/735) live with both dogs and cats (Table 1).

### 3.2. Clinical Distribution of CAD

#### 3.2.1. Temporal Trends in CAD Diagnosis

The clinical distribution of the 735 dogs with the disease is shown in Table 2, Table 3 and Table 4, where it can be seen that the annual distribution of the 735 dogs diagnosed with canine atopic dermatitis (CAD) between 2018 and 2025 shows a marked temporal variation in the number of cases treated. During the first years of the study period (2018–2020), the number of diagnoses was low and relatively stable representing only 10.75% of the total cases. This behavior could be related to lower clinical suspicion, underdiagnosis, or an incipient registration of the disease in the early years. From 2021 onwards, there was a sharp increase in the number of diagnoses, reaching 20.68% of the total, a trend that remained high in subsequent years (2022–2024). The year 2024 had the highest proportion of cases (24.22%), suggesting greater awareness of the disease, improvements in diagnostic criteria, increased patient referral, or a real increase in the frequency of CAD in the population served. In 2025, there was a relative decrease (8.3%), which should be interpreted with caution, as it corresponds to a partially evaluated year, limiting direct comparability with previous full years. Overall, these results reflect a growing trend in the identification of CAD beginning 2021 onwards, although the data do not represent the national epidemiological situation, but only the canine population evaluated in this study, predominantly from the province of Pichincha.

#### 3.2.2. Geographic and Environmental Distribution

The geographic distribution of the 735 dogs diagnosed with CAD shows a clear predominance of cases from urban areas, which accounted for 88.84% of the total, compared to 11.16% from rural areas, with statistically significant differences (*p*-value: <0.0001). This marked difference suggests that CAD is diagnosed more frequently in urban environments within the population evaluated. This pattern may be influenced by several non-exclusive factors, including greater access to specialized veterinary services in urban areas, greater awareness of chronic dermatological diseases among guardians, and a greater likelihood of consultation in the event of persistent clinical signs, such as pruritus or skin lesions. Furthermore, in urban environments, dogs may be more exposed to domestic environmental allergens (dust mites, air pollutants, cleaning products), which have been associated with the exacerbation of atopic diseases. Conversely, the lower proportion of cases from rural areas could reflect underdiagnosis, limited access to specialized veterinary care, or differences in consultation patterns, rather than a true lower incidence of the disease.

#### 3.2.3. Demographic Characteristics

In terms of sex, 52.38% (385/735) were male and 47.62% (350/735) were female, with a significant difference (*p*-value: 0.00019), indicating that the sex distribution is not homogeneous in the population studied. Likewise, 74.97% (551/735) belong to dogs classified according to their morphological characteristics within a defined breed, as detailed in Table 4, while 25.03% (184/735) belong to mixed-breed animals. The French Bulldog was the most predominant breed with CAD. However, the descriptive analysis of the distribution of breeds by sex (*p*-value: 0.006841), excluding mixed-breed dogs, reveals some interesting trends. Although in general, most breeds show a relatively balanced distribution between males and females, some specific breeds show a marked predominance of one sex. For example, breeds with a predominance of males are the French Bulldog (42 males versus 28 females), American Bully (12 males versus 4 females), Beagle (14 males and 7 females), and West Highland White Terrier (17 males to 5 females). The Shiba Inu breed is notable among breeds with a predominance of females (31 females to 21 males). These results may reflect selection trends on the part of owners or specific breeding practices depending on the breed.

#### 3.2.4. Clinical Manifestations

With regard to parasite control, 58.10% (427/735) of the animals were dewormed internally and 53.33% (392/735) externally (Table 2), with no significant differences between these variables. Regarding the first symptom reported by the guardians, 80.27% (590/735) indicated pruritus, 9.79% (72/735) indicated erythema, 5.31% (39/735) indicated alopecia, 3.67% (27/735) indicated otitis, and 0.96% (7/735) indicated bad odor, with no significant differences between them. The only statistically significant difference was observed between the association of the year and the main symptom (*p*-value: <0.0001), as pruritus was the predominant clinical symptom of CAD throughout the study period (2018–2025), accounting for the majority of annual records, with a maximum of 151 cases in 2024. Erythema was identified as the second most frequent sign, although with a much lower prevalence (maximum of 26 cases in 2021). Alopecia and otitis occurred in smaller proportions and intermittently.

When analyzing the degree of pruritus according to the Pruritus Visual Analog Scale (pVAS) [33,34], 22.86% (168/735) had a score of 8, 15.92% (117/735) had a score of 10, 12.11% (89/735) had grade 7, and 11.84% (87/735) had grade 6 (Table 2). When categorizing pruritus severity using the Pruritus Visual Analog Scale (pVAS), most dogs presented moderate-to-severe pruritus, with a clear predominance of scores between 7 and 10, confirming that the majority of patients were evaluated at advanced clinical stages. Additionally, a Composite Severity Distribution Score (CSDS) was calculated, integrating pruritus intensity and lesion distribution. The CSDS analysis revealed that a high proportion of dogs were classified within moderate-to-severe categories, reinforcing the clinical burden of CAD in the studied population and supporting the pVAS findings. However, the severity of pruritus in dogs with atopic dermatitis showed a significant variation (*p*-value: <0.0001) between the study periods, with the early years (2018–2020) mainly recording cases with severe pruritus (grades 6–10). From 2021 onwards, there was a notable increase in diagnoses at all levels, with grade 8 being the most frequent (36 cases in 2021, 38 in 2022, and 37 in 2024). In 2023, however, there was an increase in cases with moderate pruritus (grades 3–6). Overall, severe grades (7–10) predominated throughout the period, reflecting that most patients came to the clinic with advanced clinical symptoms.

Clinically, the most affected area of the body was the abdomen, with 24.49% (180/735), followed by the forelimbs with 17.68% (130/735), ears with 15.65% (115/735), and hind legs with 14.68% (108/735), with no significant differences between them. The anatomical distribution of lesions showed significant variations (*p*-value: <0.0001) throughout the study period (2018–2025). The abdomen and forelimbs were the most frequently affected regions in the early years, with peaks in 2021 (49 and 38 cases, respectively). However, from 2023 onwards, a change in the clinical pattern was evident, with an increase in cases in the ears (43 in 2024), hind limbs (67 in 2024), and eyelids (20 in 2024). This transition suggests that, although the classic areas (abdomen and forelimbs) remain relevant, there is a recent trend toward greater involvement of areas such as ears, eyelids, and hind limbs. The comparison between rural and urban areas showed marked differences in the anatomical distribution of lesions (*p* value: <0.0001), as in rural areas the abdomen was predominantly affected (32 cases), followed by the forelimbs (18), and the dorsal region (8). In contrast, in urban areas, in addition to the abdomen (148 cases), high frequencies were observed in the hind limbs (129) and ears (109), suggesting a broader and more severe clinical pattern. These results indicate that, while in rural areas atopic dermatitis mainly affects the ventral and anterior areas, in urban areas it manifests more frequently in the hind limbs and auricles, which could be related to environmental differences, exposure to allergens, or diagnostic access factors. Furthermore, the integration of CSDS with anatomical distribution showed that dogs with higher CSDSs were more likely to present lesions in multiple body regions, particularly ears, hind limbs, and abdomen, suggesting a more generalized disease pattern in severe cases.

#### 3.2.5. Environmental and Lifestyle Factors

The diet of CAD-positive animals consisted of mixed feed in 41.91% (308/735) of cases, super-premium feed in 31.84% (234/735), premium feed in 16.6% (122/735), and homemade feed in 4.76% (355/735), with statistically significant differences (*p*-value: <0.0001) between them.

Likewise, taking into account the age of the animals, statistically significant differences were found (*p*-value: <0.0001), with 84.22% (619/735) being adults, 10.20% (75/735) being puppies, and only 5.58% (41/735) being geriatric (Table 2), with a mean age of 59.66 months or 5 years, the youngest animal being 4 months old and the oldest being 193 months or 16 years old (Table 3). The distribution of cases by age group varied significantly between 2018 and 2025 (*p*-value: <0.0001), with adult dogs accounting for the majority of diagnoses throughout the period, with a notable increase in 2021–2024, reaching a peak of 150 cases in 2024. In contrast, geriatric dogs were the minority, although they showed a progressive increase, especially in 2023 (8 cases) and 2024 (20 cases). Puppies fluctuated to a lesser extent, with peaks in 2021 (21 cases) and 2023 (17 cases). These differences reflect a significant variation in the presentation of the disease over time according to age group. The analysis of age in months according to the most affected body area showed statistically significant differences (*p*-value: 0.03581) after analysis. The post hoc analysis found that dogs with ear lesions were significantly younger than those with abdominal lesions (*p*-value: 0.016), but significantly older than those with lesions on the front limbs (*p*-value: 0.034) and mouth (*p*-value: 0.035). No statistically significant differences were observed between the other comparisons. The Kruskal–Wallis test determined significant differences (*p*-value: 0.03637) between these variables. Subsequently, the post hoc analysis showed that these differences were between otitis and alopecia (*p* value: 0.0496), indicating that dogs with otitis as the main clinical sign were more prevalent among younger dogs than those with alopecia; no significant differences were noted in other comparisons; however, a trend towards older age was observed in dogs with pruritus relative to those with otitis (*p*-value: 0.0595).

**Figure 3:**
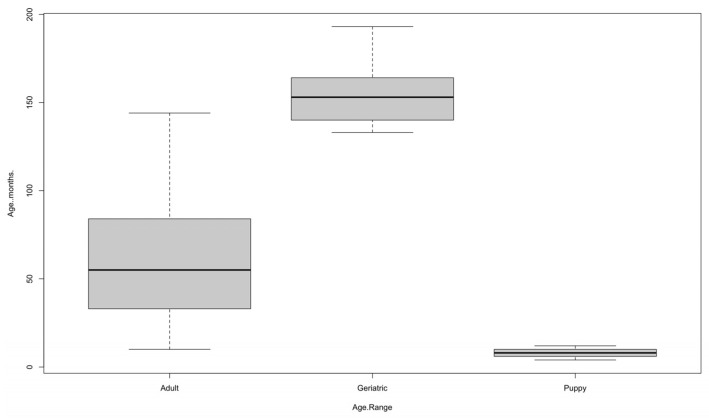
Boxplot of the relationship between yearly age and age in months.

In Figure 3, it can be seen that the age variable [11] shows an expected pattern, as it is clear that very young puppies are homogeneous, adults are more heterogeneous, and geriatric dogs are concentrated in higher age ranges; adults are the group with the greatest dispersion, while puppies and geriatric dogs show greater homogeneity.

In the case of puppies, the median is around 6 months, with a narrow interquartile range of approximately 5 to 8 months. No atypical values are observed, indicating that the group is fairly homogeneous. In adults, the median is around 55 months (≈4.5 years). The interquartile range varies approximately between 35 and 85 months. The group shows greater dispersion than puppies and includes extreme values reaching up to about 150 months. This shows significant variability in the age of diagnosed adult dogs. Geriatric animals have a median of 155 months (almost 13 years), with an interquartile range from 145 to 165 months. Although the range is relatively narrow, the values reach up to about 190 months (16 years). This reflects that, within this group, most dogs are concentrated in advanced ages, as expected, but still illustrating some variability (Figure 3).

In terms of body weight, the lowest value is 1.1 kg and the highest is 53.4 kg, with an average of 16.14 kg. In both cases, the coefficient of variation is greater than 60, meaning that the population studied is very heterogeneous in terms of age and body weight (Table 3), as both are very young puppies and geriatric animals of different breeds, and therefore different sizes and weights have been diagnosed. Statistical differences were determined between the body weight of the animals and their sex, with males having a significantly higher average weight than females. This finding indicates that sex is a relevant factor to consider when analyzing weight variations in the population studied.

As shown in Table 2, 551 of the 735 animals diagnosed with CAD are purebred, with statistically significant differences between breeds (*p*-value: 0.01), where the most common breed is the French Bulldog with 12.7% (70/566), followed by the Standard Schnauzer with 9.8% (54/566), Shih Tzu with 9.44% (52/566), Yorkshire Terrier with 4.36% (24/566), and German Shepherd Dog, West Highland White Terrier, and Golden Retriever with 4.00% (22/566) each (Table 4).

**Figure 4:**
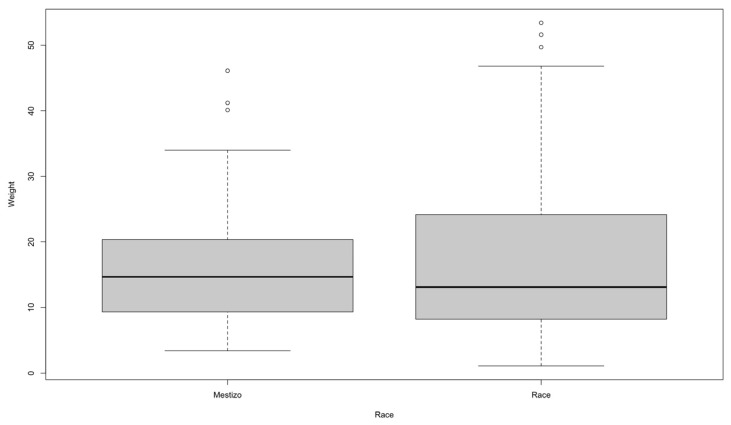
Boxplot of the relationship between race and body weight.

Figure 4 shows that mixed-breed dogs have a median weight of around 15 kg, with an approximate interquartile range between 10 and 20 kg. Outliers are above 35 kg, reaching up to about 45 kg. This indicates that most mixed-breed dogs are concentrated in a medium weight range, although there are individuals that are considerably heavier than the central tendency of the group. In purebred dogs, the median is slightly lower, close to 13 kg, with a wider IQR (approximately between 8 and 23 kg). In addition, there are outliers above 50 kg. This reflects a greater dispersion in the weight of purebred animals compared to mixed breeds, evidencing a wider variability within the group. When comparing both groups, it can be seen that mixed breeds have a higher median weight, but with less variability than purebreds. In contrast, purebred animals show greater heterogeneity, with some specimens much heavier than the group average.

#### 3.2.6. Multivariate Analysis of Factors Associated with CAD Severity

A multivariate logistic regression model was performed to identify factors associated with increased disease severity (based on CSDS classification). The analysis revealed that urban origin was significantly associated with higher odds of moderate-to-severe CAD (OR > 1; *p* < 0.05). Similarly, adult age group and purebred status were also identified as significant predictors of increased severity (*p* < 0.05). In contrast, variables such as sex, deworming status, and cohabitation with other animals were not significantly associated with disease severity in the adjusted model (*p* > 0.05). The model demonstrated adequate goodness-of-fit and allowed identification of independent risk factors influencing CAD clinical expression in Ecuador, highlighting the multifactorial nature of the disease in the studied population (Table 5).

## 4. Discussion

No published studies describing the epidemiological and clinical characteristics of canine atopic dermatitis (CAD) in Ecuador have been found; therefore, this study constitutes an unprecedented contribution in this context. The results obtained are based exclusively on the canine population evaluated in this research. In this sense, of the 735 dogs diagnosed with CAD between January 2018 and July 2025, with regard to geographical distribution, the vast majority belonged to the province of Pichincha (97.55%), being raised in a cold climate and coming mainly from urban areas (88.84%). Likewise, most of these dogs, as part of their daily routine, had some type of contact with grass (64.35%), with the vast majority living in houses with lawns (58.78%) where they coexist with other dogs (48.16%) or where they are the only pet (46.94%).

Grass pollen, dust, mites, fungi, mold, insects such as ants and bees, ectoparasites such as fleas and ticks, the geographical location where the animal is found, lifestyle, as well as contact with the outside world, i.e., whether the animal lives in urban areas, especially indoors with exposure to tobacco smoke and large factories, are predisposing factors that can facilitate the development of CAD [5,40,41]. On the other hand, increased hygiene and cleanliness levels associated with current lifestyles and high human density favor a decrease in the diversity of pathogens and infectious agents to which the animal is exposed. For this reason, sufficient antibodies are not generated to prevent the development of allergies in the individual [5,17].

Environmental and geographic conditions may influence the clinical presentation and diagnosis of canine atopic dermatitis. In the present study, the predominance of cases from urban environments and cold climates may reflect both environmental exposure to indoor allergens such as house dust mites and differences in access to specialized veterinary care. Similar patterns have been described in epidemiological studies conducted in Europe and Australia, where urban environments and lifestyle factors have been associated with increased diagnosis of allergic skin diseases in companion animals [3,42]. Importantly, the multivariate analysis performed in this study supports this observation, identifying urban origin as an independent factor associated with increased CAD severity; thus, suggesting that environmental exposure in urban settings not only influences diagnosis frequency but also disease expression.

The epidemiological patterns observed in the present study provide additional insights into the clinical distribution of canine atopic dermatitis in the studied population. Differences in the frequency of cases according to breed, age, and sex have been reported in several epidemiological studies, suggesting that both genetic predisposition and environmental factors may influence disease expression. In our study, the distribution of cases among different breeds and age groups is consistent with previous reports indicating that CAD frequently affects young to middle-aged dogs and may show breed predispositions. Additionally, the anatomical distribution of lesions observed in this study corresponds to the typical clinical presentation described in the literature, particularly involving areas such as the paws, ears, face, and ventral regions of the body. These findings are comparable with those reported in epidemiological studies conducted in Europe and North America, which highlight the multifactorial nature of CAD and the importance of both host and environmental factors in the development and clinical expression of the disease [42,43,44].

Furthermore, the integration of lesion distribution with the Composite Severity Distribution Score (CSDS) allowed a more comprehensive characterization of disease severity, demonstrating that dogs with higher severity scores tend to present a more generalized distribution of lesions, particularly affecting ears, hind limbs, and ventral regions. This finding reinforces the concept that CAD severity is not only determined by pruritus intensity but also by the extent of anatomical involvement.

Regarding the clinical distribution of the 735 dogs with the disease, 74.97% are purebred and 25.03% are mixed breed. Among purebred animals, the French Bulldog was the most common breed, accounting for 12.7%, followed by the Standard Schnauzer, Shih Tzu, Yorkshire Terrier, German Shepherd Dog, West Highland White Terrier, and Golden Retriever. In a study conducted in Australia, the Pug and Bichon Frise were determined to be the breeds with the highest frequency of CAD [43]. Scientific evidence has suggested that several pure breeds, such as the Golden Retriever, Labrador Retriever, German Shepherd Dog, West Highland White Terrier, French Bulldog, Boxer, Pug, and Beagle are predisposed to developing canine atopic dermatitis due to genetic mutations that vary between breeds, although no common markers have been found. It is also important to mention that certain animals carry the CAD genes (*FLG*, *FLG*, *SPINK5*, *CLDN1*, *DSG1*, *IL4*, *IL13*, *IL31*, *IL5*, *TNF*, *TSLP*, *STAT6*, *TLR2*, or *TLR4*) but do not develop the disease throughout their lives [5,12,17]. Retrospective studies conducted in Brazil show that mixed breeds are also predisposed to developing CAD, although they are clearly influenced by the geographical area where the animal lives. Among them are Poodles, Shih Tzus, Shar-peis, Cocker Spaniels, Dalmatians, English Bulldogs, Setters, Boston Terriers, Cairn Terriers, and Scottish Terriers [5,12,17]. The multivariate model further confirmed that purebred dogs had a higher probability of presenting more severe forms of CAD, supporting the role of genetic susceptibility described in previous studies.

In our study, 52.38% of the animals were male and 47.62% were female, with 84.22% being adults, with an average age of 59.66 months (5 years) and a body weight between 1.1 kg and 53.4 kg (average 16.14 kg). These data are similar to those reported by several authors, who indicate that CAD is more prevalent in males (especially neutered males) between 4 and 6 months and 3 years of age [5,17]. However, sex was not identified as a significant predictor of disease severity in the multivariate analysis, suggesting that although distribution differences exist, sex may not independently influence clinical progression.

With regard to parasite control, 58.10% have been dewormed internally and 53.33% externally. This factor is important because, although ectoparasites are not considered to cause CAD, they can increase the sensation of pruritus (especially fleas) and complicate the condition, so controlling them is an appropriate prevention strategy [44]. Similarly, another study mentions a possible protective effect of intestinal parasites, helminths, such as *Toxocara canis*, against CAD induced by dust mites, and their power to regulate the immune response [5,42]. Interestingly, parasite control variables were not significantly associated with CAD severity in the adjusted model, indicating that their role may be more related to symptom exacerbation rather than disease progression itself.

The first symptom reported by the dogs’ owners is pruritus (80.27%), and the remaining were erythema, alopecia, otitis, and bad odor. In different studies, pruritus is identified as the most distinctive clinical manifestation of the disease, prompting animals to be taken to the veterinarian [7,8,15]. It is known that CAD is triggered by hereditary genetic modifications that stimulate an abnormal immune response due to an exacerbation in IgE production. This results in alterations in the stratum corneum of the skin, facilitating the penetration of exogenous proteins into the animal’s epidermis, thereby damaging the lipid layer of the cutaneous barrier. Consequently, this leads to greater transepidermal water loss and a reduction in ceramides, generating hypersensitivity to exposure to certain allergens, irritants, and microorganisms, which incites skin inflammation and subsequent lesions due to alterations in the animal’s skin microbiota, ultimately resulting in pruritus [15,35,45].

Regarding the degree of pruritus, 22.86% presented grade 8, 15.92% presented grade 10, 12.11% (89/735) presented grade 7, and 11.84% (87/735) presented grade 6 (Table 2). From a clinical perspective, the predominance of high pVAS scores (7–10) indicates that most animals were evaluated at moderate-to-severe stages of the disease. This finding is consistent with referral bias in specialized centers, where patients are more likely to present advanced clinical signs. Moreover, the use of pVAS in combination with CSDS in this study represents a methodological improvement, as it allows not only quantification of pruritus intensity but also integration with lesion distribution, providing a more robust and clinically meaningful assessment of disease severity.

Clinically, the most affected area of the body was the abdomen, with 24.49% of cases, followed by the forelimbs, ears, and hind paws. Among the main intrinsic factors responsible for the inflammatory process and enhancing the animal’s immune response include Langerhans cells and skin dendritic cells, which capture and present the antigen; B and T cells that produce cytokines, many of which are activated through the JAK-STAT pathway, with interleukin 31 triggering pruritus and skin inflammation; and mast cells found in the animals’ skin, which are classified as producers of inflammatory mediators. The relative importance of these cells remains undetermined, yet their interaction is known to culminate in the clinical manifestation of the pathology [46]. Likewise, studies in dogs with CAD have found that activation of Toll-like receptors and PAR-2 in keratinocytes produces cytokines and chemokines, which are responsible for the symptomatology, although there are many inconsistent results complicating the study of the disease [15,47,48]. This pruritus can worsen the condition since it can cause acral lick dermatitis, which can lead to ulcers in dogs [46].

The diets of animals tested positive for CAD were mixed for 41.91% (308/735), super-premium for 31.84% (234/735), premium for 16.6% (122/735), and homemade for 4.76% (355/735). This high percentage of animals that consume a diet based on super premium food or a mixed diet could be due to the fact that these are dogs with suspected food allergy, whose diet is of utmost importance for the treatment of CAD [49]. Previous research suggests that the relationship between the cutaneous microbiota and CAD begins during the animal’s puppy stage, since proper feeding based on probiotics, nutrients, and colostrum within the first 16 h of life helps to considerably reduce the presentation of the disease, thus developing a healthy intestinal microbiota [7,8,50].

Although diet showed significant differences in the descriptive analysis, it was not retained as a significant predictor in the multivariate model, suggesting that dietary patterns may be influenced by clinical management decisions rather than acting as primary risk factors.

The importance of this study also lies in the fact that CAD has many clinical and pathophysiological similarities with human atopic dermatitis, which worldwide affects on between 15 and 20% of children and between 3 and 5% of the adult population [51]. Given the absence of a definitive cure and the adverse long-term effects associated with various treatments, it is essential to identify the factors contributing to CAD. Additionally, skin and coat care, along with the exploration of preventive and therapeutic alternatives, is crucial, particularly in promoting preventive medicine to mitigate CAD flare-ups in dogs [28,52], which can be applied in veterinary practice in Ecuador.

In addition, the incorporation of multivariate analysis and composite severity indexes strengthens the epidemiological value of this study, addressing limitations commonly identified in descriptive studies and responding to current recommendations for more integrative analytical approaches in veterinary dermatology research.

Overall, these findings highlight the multifactorial nature of CAD in Ecuador, where environmental, demographic, and possibly genetic factors interact to influence both disease occurrence and severity, providing a foundation for future prospective studies and targeted prevention strategies.

### Limitations

A limitation of this study is that detailed information regarding the specific allergens identified through intradermal testing was not consistently recorded in the clinical database. As a result, it was not possible to perform a statistical analysis of allergen sensitization patterns in the studied population. Future studies evaluating the prevalence and distribution of specific environmental allergens in dogs with canine atopic dermatitis (CAD) in Ecuador would provide valuable information for improving diagnostic approaches and developing region-specific management strategies. Additionally, due to the retrospective nature of the study (2018–2025), standardized and validated clinical scoring systems such as CADESI or CADLI were not routinely recorded at the time of consultation. This represents an important limitation, as it precluded the use of gold-standard lesion severity indices and limited direct comparability with other studies. In this context, the Composite Clinical Severity and Distribution Score (CSDS) was developed as a pragmatic and methodologically transparent alternative to integrate available clinical information; however, it does not replace validated scoring systems and should be interpreted with caution. Furthermore, the study population was predominantly derived from a single referral veterinary center located in an urban area (Quito), which may introduce selection bias and limit the generalizability of the findings to the broader Ecuadorian canine population, particularly in rural or underrepresented regions. Although the Composite Severity Distribution Score (CSDS) provided an integrative measure of disease severity, its use represents an exploratory approach that requires external validation in independent populations to confirm its reproducibility and clinical applicability. Finally, potential contradictory variables such as prior treatments, environmental exposure intensity, and genetic background were not fully controlled, which may have influenced the observed associations. Future prospective and longitudinal studies are warranted to validate these findings and further elucidate causal relationships.

## 5. Conclusions

In conclusion, it was found that CAD in Ecuador mainly affects adult, purebred, male dogs, with a predominance of severe pruritus and lesions on the abdomen and limbs. The urban distribution and cold climate suggest an influence of environmental factors and differential access to veterinary care. Significant associations were evidenced between age, sex, breed, weight, and lesion location, which are useful to guide clinical management. Significantly, the incorporation of the Pruritus Visual Analog Scale (pVAS) and the Composite Severity Distribution Score (CSDS) allowed a more comprehensive assessment of disease severity, demonstrating that most patients present moderate-to-severe clinical forms with widespread lesion distribution. Furthermore, the multivariate analysis identified urban origin, adult age, and purebred status as independent factors associated with increased disease severity, highlighting the multifactorial nature of CAD and the interaction between environmental and host-related variables. The systematic characterization presented constitutes an unprecedented database for future research, prevention strategies, and rational selection of treatments in the Ecuadorian canine population. Overall, these findings provide clinically relevant evidence to support early diagnosis, improve severity stratification, and guide targeted therapeutic and preventive approaches in veterinary practice, while establishing a robust baseline for future epidemiological and longitudinal studies in the region.

## Figures and Tables

**Table 1:** Demographic characteristics of all patients diagnosed with CAD (*n* = 735).

Variable	Category	Number (of 735)	Percentage (%)
Year	2018	25	3.4
	2019	26	3.54
	2020	28	3.81
	2021	152	20.68
	2022	139	18.91
	2023	126	17.14
	2024	178	24.22
	2025	61	8.3
Province	Pichincha	717	97.55
	Chimborazo	1	0.14
	Carchi	1	0.14
	Imbabura	2	0.27
	Manabí	4	0.54
	Orellana	1	0.14
	Santo Domingo	4	0.54
	Tungurahua	5	
Geographic Distribution	Urban	671	88.84
	Rural	84	11.16
Climate	Cold	652	86.39
	Temperate	82	10.88
	Semi-Temperate	15	2.05
	Hot	5	0.68
Contact with Grass	Yes	473	64.35
	No	262	35.65
Housing	House with grass	432	58.78
	Apartment without grass	141	19.18
	House without grass	121	16.46
	Apartment with grass	41	5.58
Other Pets	Dogs	354	48.16
	Single pet	345	46.94
	Cats	33	4.49
	Dogs and cats	3	0.41

**Table 2:** General clinical characteristics of patients affected with CAD (*n* = 735).

Variable	Category	Number (of 735)	Percentage (%)
Breed	Race	551	74.97
	Mongrel	184	25.03
Age of animals	Adult	619	84.22
	Puppy	75	10.2
	Geriatric	41	5.58
Sex	Male	385	52.38
	Female	350	47.62
Internal deworming	Yes	427	58.1
	No	308	41.9
External deworming	Yes	392	53.33
	No	343	46.67
First symptom	Pluritus	590	80.27
	Erythema	72	9.79
	Alopecia	39	5.31
	Otitis	27	3.67
	Bad odor	7	0.96
Degree of itching according to the *Pruritus Visual Analog Scale* (pVAS) [33,34]	1	23	3.13
	2	29	3.95
	3	42	5.71
	4	49	6.67
	5	74	10.05
	6	87	11.84
	7	89	12.11
	8	168	22.86
	9	57	7.76
	10	117	15.92
Most affected areas	Abdomen	180	24.49
	Hind legs	130	17.68
	Ears	115	15.65
	Front legs	108	14.68
	Back	51	6.94
	Eyelids	41	5.57
	Mouth	26	3.54
	Croup	24	3.27
	Armpits	24	3.27
	Neck	16	2.18
	Thorax	12	1.63
	Tail	8	1.1
Food	Mixed	308	41.91
	Super premium	234	31.84
	Premium	122	16.6
	Homemade	35	4.76
	Standard	27	3.67
	Barf	9	1.22

**Table 3:** CAD frequency by age and body weight.

Variable	Minimum	Maximum	Mean	Median	Standard Deviation	Coefficient of Variation
Age (months)	4	193	59.66	49.00	40.96	68.66
Weight (kg)	1.1	53.4	16.14	13.10	9.93	61.6

**Table 4:** Frequency of CAD by race.

Variable	Category	Number (of 551)	Percentage (%)
Breed name	French Bulldog	70	12.7
	Schnauzer Standard	54	9.8
	Shih Tzu	52	9.44
	Yorkshire Terrier	24	4.36
	German Shepherd Dog	22	4.00
	West Highland White Terrier	22	4.00
	Golden Retriever	22	4.00
	Labrador Retriever	20	3.63
	English Bulldog	17	3.08
	Poodle	15	2.72
	Pitbull	12	2.18
	Pug	12	2.18
	Pekingese	10	1.81
	Old English Sheepdog	7	1.27
	Other races	192	34.86

**Table 5:** Multivariate logistic regression analysis for factors associated with severe CAD (CSDS ≥ 8).

Variable	Category	OR	95% CI	*p*-Value
Age (months)	Continuous	1.01	0.99–1.02	0.082
Sex	Male vs. Female	1.12	0.85–1.48	0.412
Environment	Urban vs. Rural	1.41	0.97–2.05	0.071
Grass contact	Yes vs. No	1.78	1.22–2.60	0.003

## Data Availability

The original contributions presented in this study are included in the article/Supplementary Materials. Further inquiries can be directed to the corresponding author.

## Supplementary Materials

The following supporting information can be downloaded at https://www.mdpi.com/article/10.3390/vetsci13040351/s1. Table S1: DATA.

## References

[1] Nuttall T., Uri M., Halliwell R. (2013). Canine Atopic Dermatitis—What Have We Learned?. Vet. Rec..

[2] Almqvist C., Egmar A.-C., van Hage-Hamsten M., Berglind N., Pershagen G., Nordvall S.L., Svartengren M., Hedlin G., Wickman M. (2003). Heredity, Pet Ownership, and Confounding Control in a Population-Based Birth Cohort. J. Allergy Clin. Immunol..

[3] Marsella R. (2021). Advances in Our Understanding of Canine Atopic Dermatitis. Vet. Dermatol..

[4] Banovic F. (2025). Updated Insights into the Molecular Pathogenesis of Canine Atopic Dermatitis. Vet. Dermatol..

[5] Hensel P., Santoro D., Favrot C., Hill P., Griffin C. (2015). Canine Atopic Dermatitis: Detailed Guidelines for Diagnosis and Allergen Identification. BMC Vet. Res..

[6] Bizikova P., Santoro D., Marsella R., Nuttall T., Eisenschenk M.N.C., Pucheu-Haston C.M. (2015). Review: Clinical and Histological Manifestations of Canine Atopic Dermatitis. Vet. Dermatol..

[7] Arcique M.A., Bajwa J. (2020). Atopic Dermatitis in Humans and Dogs. Can. Vet. J..

[8] Drechsler Y., Dong C., Clark D., Kaur G. (2024). Canine Atopic Dermatitis: Prevalence, Impact, and Management Strategies. Vet. Med. Res. Rep..

[9] Kurmann S., Coelho M.A., David Palma M., Díaz L., Castellá G., Cabañes F.J., Heitman J., LeibundGut-Landmann S., Muchaamba F. (2025). Fourier Transform Infrared Spectroscopy Enables Rapid Species Discrimination across Malassezia and Strain-Level Typing in *M. Pachydermatis*. bioRxiv.

[10] Santoro D., Saridomichelakis M., Eisenschenk M., Tamamoto-Mochizuki C., Hensel P., Pucheu-Haston C. (2024). Update on the Skin Barrier, Cutaneous Microbiome and Host Defence Peptides in Canine Atopic Dermatitis. Vet. Dermatol..

[11] Forster S., Boegel A., Despa S., Trout C., King S. (2025). Comparative Efficacy and Safety of Ilunocitinib and Oclacitinib for the Control of Pruritus and Associated Skin Lesions in Dogs with Atopic Dermatitis. Vet. Dermatol..

[12] Nuttall T.J., Marsella R., Rosenbaum M.R., Gonzales A.J., Fadok V.A. (2019). Update on Pathogenesis, Diagnosis, and Treatment of Atopic Dermatitis in Dogs. J. Am. Vet. Med. Assoc..

[13] Gugliandolo E., Palma E., Cordaro M., D’Amico R., Peritore A.F., Licata P., Crupi R. (2020). Canine Atopic Dermatitis: Role of Luteolin as New Natural Treatment. Vet. Med. Sci..

[14] Boerngen K., Patel Y., Pittorino M., Toutain C.E. (2026). Pharmacokinetics of Ilunocitinib, a New Janus Kinase Inhibitor, in Dogs. J. Vet. Pharmacol. Ther..

[15] Bensignor E. (2010). La Dermatite Atopique Canine. Bull. Acad. Natl. Med..

[16] Gonçalves M., Fernandes B., Alves S.P., Pereira H., Prego M.T., Lourenço A.M. (2026). Preliminary Measurement of Faecal Short-Chain Fatty Acids in Dogs with Canine Atopic Dermatitis. Vet. Dermatol..

[17] Marsella R. (2012). An Update on the Treatment of Canine Atopic Dermatitis. Vet. Med. Res. Rep..

[18] Schumann J., Basiouni S., Gück T., Fuhrmann H. (2014). Treating Canine Atopic Dermatitis with Unsaturated Fatty Acids: The Role of Mast Cells and Potential Mechanisms of Action. J. Anim. Physiol. Anim. Nutr..

[19] Miranda-Cortés A., Mota-Rojas D., Crosignani-Outeda N., Casas-Alvarado A., Martínez-Burnes J., Olmos-Hernández A., Mora-Medina P., Verduzco-Mendoza A., Hernández-Ávalos I. (2023). The Role of Cannabinoids in Pain Modulation in Companion Animals. Front. Vet. Sci..

[20] Kaneki M., Ohira C., Takahashi M., Fukuyama T. (2026). Preventive Effect of Bentonite against Pyoderma via Direct Binding Capability of Staphylococci. PLoS ONE.

[21] Bell A., Nakamura Y., Langley R., Hardcastle M., Katayama Y., Middleditch M. (2026). Staphylococcus Pseudintermedius Isolated from Atopic Dogs with Pyoderma Induces Mast Cell Degranulation. N. Z. Vet. J..

[22] Nederveld S.M., Krautmann M.J., Mitchell J. (2025). Safety of the Selective JAK1 Inhibitor Oclacitinib in Dogs. J. Vet. Pharmacol. Ther..

[23] Wichtowska A., Olejnik M. (2025). Anti-Cytokine Drugs in the Treatment of Canine Atopic Dermatitis. Int. J. Mol. Sci..

[24] Pinto M.S.d.N., Gil S.J.R.C.A., Ramió-Lluch L., Schmidt V.M., Pereira H.M.L., Fernandes B.A.P., Camões A.F.B., Morais-Almeida M., São Braz B.M.F.F., Marto J.M. (2024). Challenging the Norm: Epicutaneous Immunotherapy for Canine Atopic Dermatitis. Allergy.

[25] DeBoer D.J. (2017). The Future of Immunotherapy for Canine Atopic Dermatitis: A Review. Vet. Dermatol..

[26] Mueller R.S. (2023). A Systematic Review of Allergen Immunotherapy, a Successful Therapy for Canine Atopic Dermatitis and Feline Atopic Skin Syndrome. J. Am. Vet. Med. Assoc..

[27] Kim M.S., Kong D., Han M., Roh K., Koo H., Lee S., Kang K.-S. (2023). Canine Amniotic Membrane-Derived Mesenchymal Stem Cells Ameliorate Atopic Dermatitis through Regeneration and Immunomodulation. Vet. Res. Commun..

[28] Santoro D. (2019). Therapies in Canine Atopic Dermatitis. Vet. Clin. N. Am. Small Anim. Pract..

[29] DeBoer D.J. (2004). Canine Atopic Dermatitis: New Targets, New Therapies. J. Nutr..

[30] Dubrac S., Combarros D., Oláh A., Cadiergues M.-C., Simon M. (2026). Recommendations on in Vitro Models for Canine Atopic Dermatitis. J. Investig. Dermatol..

[31] Olivry T., DeBoer D.J., Favrot C., Jackson H.A., Mueller R.S., Nuttall T., Prélaud P. (2015). Treatment of Canine Atopic Dermatitis: 2015 Updated Guidelines from the International Committee on Allergic Diseases of Animals (ICADA). BMC Vet. Res..

[32] Favrot C., Steffan J., Seewald W., Picco F. (2010). A Prospective Study on the Clinical Features of Chronic Canine Atopic Dermatitis and Its Diagnosis. Vet. Dermatol..

[33] Hill P.B., Lau P., Rybnicek J. (2007). Development of an Owner-assessed Scale to Measure the Severity of Pruritus in Dogs. Vet. Dermatol..

[34] Olivry T., Marsella R., Iwasaki T., Mueller R. (2007). Validation of CADESI-03, a Severity Scale for Clinical Trials Enrolling Dogs with Atopic Dermatitis. Vet. Dermatol..

[35] Olivry T., Saridomichelakis M., Nuttall T., Bensignor E., Griffin C.E., Hill P.B. (2014). Validation of the Canine Atopic Dermatitis Extent and Severity Index (CADESI)-4, a Simplified Severity Scale for Assessing Skin Lesions of Atopic Dermatitis in Dogs. Vet. Dermatol..

[36] Dharmarajan S., Lee J., Izem R. (2019). Sample Size Estimation for Case-crossover Studies. Stat. Med..

[37] IPCC (2006). 2006 IPCC Guidelines for National Greenhouse Gas Inventories.

[38] Harvey N.D. (2021). How Old Is My Dog? Identification of Rational Age Groupings in Pet Dogs Based Upon Normative Age-Linked Processes. Front. Vet. Sci..

[39] Strzok E., Torres S.M.F., Koch S.N., Rendahl A.K. (2022). Validation of the 0–10 Verbal Numeric Scale for Assessment of Pruritus Severity in Dogs. Vet. Dermatol..

[40] Gentry C.M. (2025). Updates on the Pathogenesis of Canine and Feline Atopic Dermatitis. Vet. Clin. N. Am. Small Anim. Pract..

[41] Bizikova P., Pucheu-Haston C.M., Eisenschenk M.N.C., Marsella R., Nuttall T., Santoro D. (2015). Review: Role of Genetics and the Environment in the Pathogenesis of Canine Atopic Dermatitis. Vet. Dermatol..

[42] Hensel P., Saridomichelakis M., Eisenschenk M., Tamamoto-Mochizuki C., Pucheu-Haston C., Santoro D. (2024). Update on the Role of Genetic Factors, Environmental Factors and Allergens in Canine Atopic Dermatitis. Vet. Dermatol..

[43] Mazrier H., Vogelnest L.J., Thomson P.C., Taylor R.M., Williamson P. (2016). Canine Atopic Dermatitis: Breed Risk in Australia and Evidence for a Susceptible Clade. Vet. Dermatol..

[44] Saridomichelakis M.N., Olivry T. (2016). An Update on the Treatment of Canine Atopic Dermatitis. Vet. J..

[45] Gentry C.M. (2025). Updates on the Pathogenesis of Canine Atopic Dermatitis and Feline Atopic Skin Syndrome: Part 2, the Skin Barrier, the Microbiome, and Immune System Dysfunction. Vet. Clin. N. Am. Small Anim. Pract..

[46] Shumaker A.K. (2019). Diagnosis and Treatment of Canine Acral Lick Dermatitis. Vet. Clin. N. Am. Small Anim. Pract..

[47] Tamamoto-Mochizuki C., Santoro D., Saridomikelakis M.N., Eisenschenk M.N.C., Hensel P., Pucheu-Haston C. (2024). Update on the Role of Cytokines and Chemokines in Canine Atopic Dermatitis. Vet. Dermatol..

[48] Pucheu-Haston C.M., Santoro D., Bizikova P., Eisenschenk M.N.C., Marsella R., Nuttall T. (2015). Review: Innate Immunity, Lipid Metabolism and Nutrition in Canine Atopic Dermatitis. Vet. Dermatol..

[49] Eisenschenk M.N.C. (2025). The Role of Diet, Nutrition, and Supplements in Canine Atopic Dermatitis. Vet. Clin. N. Am. Small Anim. Pract..

[50] Pacheco R.C.F., Lins L.F., De Brito L.P., De Andrade Calaca P.R., Porto A.L.F., Cavalcanti M.T.H. (2025). Probiotics as an Adjunct in the Treatment of Canine Atopic Dermatitis: A Systematic Review and Meta-Analysis of Studies in Dogs. J. Vet. Med. Sci..

[51] Vogelnest L. (2021). Canine Atopic Dermatitis: A Common, Chronic and Challenging Dermatosis. Vet. Rec..

[52] Olivry T., Banovic F. (2019). Treatment of Canine Atopic Dermatitis: Time to Revise Our Strategy?. Vet. Dermatol..

